# Editorial: Insight into healthcare-associated fungal infections

**DOI:** 10.3389/fcimb.2026.1843184

**Published:** 2026-04-20

**Authors:** Nour Fattouh, Sadri Znaidi, Roy A. Khalaf

**Affiliations:** 1 Department of Biology, Saint George University of Beirut, Beirut, Lebanon; 2 Department of Biology, Memorial University of Newfoundland, St. John’s, NL, Canada; 3 Institut Pasteur de Tunis, University of Tunis El Manar, Laboratoire de Microbiologie Moléculaire, Vaccinologie et Développement Biotechnologique (LR16IPT01), Tunis, Tunisia; 4 Institut Pasteur, Université Paris Cité, Unité Biologie et Pathogénicité Fongiques, INRAE USC2019, Paris, France; 5 Department of Biological Sciences, Lebanese American University, Byblos, Lebanon

**Keywords:** diagnostics, epidemiology, fungal infections, surveillance, treatment

Fungal pathogens are understudied compared to other microbial pathogens but should not be underestimated since their virulence characteristics and their increasing ability of developing antifungal resistance are a threat to humans, particularly the immunocompromised. The World Health Organization has ranked fungi based on their virulence and antifungal resistance and has warned that fungal pathogens such as *Candida albicans*, *Candida auris*, and *Aspergillus fumigatus* are of critical priority as a result of their high mortality, multidrug resistance, and major hospital outbreak potential. In this editorial, we synthesize current insights into emerging fungal threats, exploring the evolving fungal infection epidemiological landscape and management. The Research Topic published original research papers and two review papers presenting fungal infections as a global health challenge and displaying some surveillance, diagnostic, and treatment strategies and hurdles.

The selected studies highlighted a rising burden of nosocomial fungal infections. The intensive care unit is a common setting for exposure to invasive fungal infections as described in Cao et al., Nascimento et al., and Zhong et al.*Candida albicans* has long been commonly isolated from patients but nowadays non *albicans Candida* species are highly being detected such as *Candida tropicalis* and *Candida parapsilosis* among others as identified in Mesquida et al., Nascimento et al., and Steixner et al. Moreover, the review of Cao et al. described the worldwide rise in *Candida auris* invasive infections and stressed on the rise of rarer fungal infections such as mucormycosis and fusariosis, the latter was also studied in the context of ophthalmic infections in immunocompetent individuals by Kang et al. In addition, Zhong et al. showed that pulmonary aspergillosis mainly caused by *Aspergillus fumigatus* could be life-threatening especially when associated with other comorbidities; in this paper a COVID19 infection supported by mechanical ventilation in Chinese hospitals.

Fungal infection diagnostic limitations remain a major concern because conventional methods sometimes have reduced sensitivity and specificity, but Cao et al. reviewed novel early detection technologies that could overcome those drawbacks for example, quick molecular detection methods such as qPCR and LAMP (loop-mediated isothermal amplification). Nascimento et al. proposed and validated the use of the MSAA (mannitol salt agar auris) selective culture medium for the purpose of *Candida auris* detection, while Zhou et al. explored how the immune markers S100A12 and PTX3 could improve early diagnosis of invasive pulmonary aspergillosis. Taken together, these efforts could help improve early and accurate detection of fungal infections which would result in a better treatment regimen choice and outcome. Indirectly, this might reduce the incidence of antifungal resistance by minimizing the use of inappropriate or empirical treatments. Kang et al. showed that combination therapy in the treatment of ophthalmic *Fusarium* spp. infections seemed to be more efficient than natamycin or voriconazole monotherapy *in vitro*. Combining the latter drugs with chlorhexidine proved to be more effective, particularly the combination of voriconazole with chlorhexidine. However, scarcity of voriconazole in Northern China led to the successful use of a natamycin/chlorhexidine combination in patients at early stages of ophthalmic fusariosis thus, highlighting the importance of adapting medical treatments to particular limitations.

To further improve early diagnostic and treatment strategies, artificial intelligence algorithms could be applied, as discussed in Cao et al. The increased prevalence of antifungal resistance and emergence of multidrug resistant fungal strains pose a serious threat, as the discovery and development of new non-cytotoxic antifungal compounds remain challenging due to the high gene orthology between fungi and humans, both being eukaryotic. For example, Cao et al.mentioned an artificial intelligence method that might overcome this challenge by aiding scientists in the design of novel antifungal peptides *de novo*.

Complementing advances in epidemiology, diagnosis, and treatment to the study of mutations involved in virulence and antifungal resistance as well as assessing genotypic diversity provide deeper insights into fungal infection dynamics and clinical outcomes. Mesquida et al. genotyped *Candida* spp. colonizing different anatomical sites of patients hospitalized in Portugal and discovered that many genotypes are also recovered from blood cultures suggesting that certain bodily niches such as the intra-abdominal cavity acted as reservoirs of candidemia. This study could raise the awareness of clinicians regarding early diagnosis through genotyping in order to adopt the ideal treatment early on to avoid candidemia. Another surveillance strategy is to identify mutations known to induce specific virulence or antifungal resistance phenotypes through a whole-genome sequencing approach as carried out by Steixner et al. on *Candida parapsilosis* isolates in Austria. This study observed a genetic diversity of *Candida parapsilosis* isolates but also identified the emergence of the ST11 lineage that previously caused epidemics. Although surveillance is important in identifying fungal infection trends, it is easily complicated by rapid genetic evolution and resistance to antifungal agents.

In conclusion, management of fungal infections still requires significant improvement but this Research Topic alongside many emerging works in the field are laying the foundation for improving diagnostics and surveillance leading to optimized prophylaxis and treatment strategies. Future work should prioritize linking clinical findings with molecular characterization of virulence and antifungal resistance. Also, clarifying the differences between resistance, heteroresistance, and tolerance to antifungal agents at the molecular and phenotypic levels, understanding microevolution in chronic infections, and integrating host-pathogen interaction studies could all be invaluable strategies in our fight against fungal infections.

